# Quality of care in hepatocellular carcinoma—A critical review

**DOI:** 10.1097/HC9.0000000000000595

**Published:** 2024-12-11

**Authors:** Jonathan Abdelmalak, John S. Lubel, Marie Sinclair, Ammar Majeed, William Kemp, Stuart K. Roberts

**Affiliations:** 1 Department of Gastroenterology, Alfred Hospital, Melbourne, Victoria, Australia; 2 School of Translational Medicine, Monash University, Melbourne, Victoria, Australia; 3 Victorian Liver Transplant Unit, Austin Health, Heidelberg, Victoria, Australia; 4 Department of Medicine, The University of Melbourne, Melbourne, Victoria, Australia

**Keywords:** care, critical, guidelines, HCC, hepatocellular carcinoma, indicators, management, narrative, quality, registry, review

## Abstract

There is significant variation in HCC management across different centers with poor adherence to evidence-based clinical practice guidelines as assessed in prior studies. In Australia, quality indicators (QIs) have recently been proposed by a multidisciplinary group of experts to help provide a framework to assess and monitor the quality of HCC care. In this review, we discuss the many areas where real-world practice deviates from evidence-based medicine, the role that QI sets play in addressing this gap, and the similarities and differences between Australian QIs and other leading treatment guidelines and QI sets from around the world. We focus on the utility of QI sets to identify opportunities for targeted improvement in the real-world clinical environment. We conclude with a discussion about the formation of a national clinical quality registry as a long-term measure to facilitate continual improvements in patient care within and across sites in order to optimize patient outcomes.

## INTRODUCTION

HCC represents a significant health care burden globally and in Australia, with an increasing number of cases and HCC-related deaths documented over the last decade, in contrast to other forms of cancer.^1^,^2^,^3^ Five-year survival of liver cancer remains poor in Australia, with a 5-year age-standardized survival rate of only 22.9%, second to only pancreatic cancer in mortality.^1^ Up to 90% of HCC occurs in the setting of underlying cirrhosis,^4^ with metabolic dysfunction–associated steatotic liver disease, alcohol-associated liver disease, chronic hepatitis B, and hepatitis C, the most common contributory diseases.^5^ The care people with HCC require is complex and necessitates an expert multidisciplinary team (MDT) or tumor board to co-ordinate management, with evidence that such care results in improved survival outcomes^6^,^7^ and widely considered the standard of care as recommended in Australian^8^ and other leading guidelines.^9^,^10^,^11^,^12^

Australian and other international guidelines for HCC management have been published^9^,^10^,^11^; however, adherence to the recommendations contained in such guidelines has been poor when assessed in prior studies,^13^,^14^,^15^,^16^ although no such studies have been done in an Australian population to date. Quality indicators (QIs) regarding HCC management have been recently published by expert multidisciplinary groups in Australia^17^ and the United States^18^—these are the first of their kind published in the peer-reviewed literature and aim to meet the need of clearly defining what constitutes quality care, particularly when treatment deviates from guideline-based practice. Monitoring of adherence to QIs allows for the identification of opportunities for improvement and optimization in health care systems, with the hope that this ultimately benefits outcomes in the long term. There is currently significant interest at the national level in implementing Clinical Quality Registries (CQR) at a greater scale across the spectrum of health care, and we believe that the best way forward for monitoring QIs in HCC is through such a CQR, which could be established nationally on a center opt-in basis. The aim of this study was to perform a critical review from an Australian perspective of the current literature around the rationale, utility, and function of monitoring QIs in HCC.

## METHODS

We performed an expansive search of the literature using PubMed, Ovid MEDLINE, Embase, and the Cochrane Central Register of Controlled Trials databases. Key search terms were “hepatocellular carcinoma,” “quality indicators,” “quality improvement,” and “guidelines” with specific search strategies tailored to the unique search software. Databases were searched from inception until 13 September 2024, with all papers available in the English language suitable for inclusion. Further literature was reviewed through the reference lists of reviewed manuscripts. Guideline recommendations were tabulated with differences highlighted according to a predefined rubric of key elements of HCC management. Specific themes around QI sets were compared.

### Overview of guidelines

Table 1 summarizes the most recent Gastroenterological Society of Australia (GESA), Japanese Society of Hepatology (JSH), Asia Pacific Association for the Study of the Liver (APASL), European Association for the Study of the Liver (EASL), and American Association for the Study of Liver Diseases (AASLD) guidelines.^8^,^9^,^10^,^11^,^12^ All themes in the guidelines reflect key standards of care in the optimal management of HCC and are discussed herein.

**TABLE 1:** Summary of HCC guidelines

Themes	GESA 2020^8^	JSH 2021^12^	APASL 2017^11^	EASL 2018^9^	AASLD 2023^10^
Surveillance in	Cirrhosis if suitable and willing to receive HCC treatment Chronic HBV at increased risk of HCC -Asian men >40 -Asian women >50 -Born in sub-Saharan Africa >20 -ATSI >50 -Family history	Extremely high risk -HBV cirrhosis -HCV cirrhosis High-risk -noncirrhotic CHB -noncirrhotic HCV -nonviral cirrhosis	Not specifically outlined	Cirrhosis -CPA or B -CPC and waitlisted for LT Noncirrhotic HBV -PAGE-B ≥10 Noncirrhotic F3 patients of any etiology subject to individualized assessment	Cirrhosis -CPA or B -Candidate of CPC and LT Noncirrhotic HBV -Man from endemic country >40 -Woman from endemic country >50 -Person from Africa at earlier age -Family history -PAGE-B ≥10 -Family history
Surveillance with	6 monthly US +/− AFP	Extremely high risk - 3–4 monthly US + tumor marker High risk - 6 monthly US + tumor marker	6 monthly US +/− AFP	6 monthly US	6 monthly US + AFP
Diagnosis with	Multiphase CT or MRI using standardized criteria Histology if not at increased risk or indeterminate lesion	Multiphase CT or MRI or CEUS or CT hepatic arteriography or CT arterioportography	Multiphase CT or MRI or CEUS	Multiphase CT or MRI or CEUS Histology if noncirrhotic	Multiphase CT or MRI using LI-RADS criteria Histology if not at increased risk or indeterminate lesion
Management framework	BCLC	JSH treatment algorithm	APASL treatment algorithm	BCLC	BCLC
Decision-making	MDT	Not discussed	MDT	MDT	MDT
Role of resection	First-line curative therapy in suitable patients (preserved liver function, sufficient liver remnant, absence of significant portal hypertension)	Recommended in all patients without extrahepatic disease and 3 or less tumors, including in patients with CP A/B cirrhosis and in portal vein invasion up to the 1st branch. Preferred in single tumors >3 cm.	Consider in all patients, including considering in patients with macrovascular invasion and multinodular disease	First-line curative therapy in noncirrhotic liver In cirrhosis, composite assessment liver function, portal hypertension, extent of hepatectomy, the expected volume of the future liver remnant, performance status, and patient's comorbidities	Treatment of choice if noncirrhotic or if compensated cirrhosis without CSPH and adequate FLR
Adjuvant therapy	Not recommended	Not mentioned	Retinoic acid and vitamin K2 are suggested.	Not recommended	Bevacizumab/Atezolizumab recommended in high-risk patients (tumor size >5 cm, more than 3 tumor, microvascular or macrovascular invasion, poor tumor differentiation)
Role of transplantation	Within transplant criteria not suitable for resection or ablation.	CPC cirrhosis within transplant criteria	CPB/C cirrhosis + HCC	Within transplant criteria not suitable for resection	Treatment of choice for transplant-eligible patients with early-stage HCC with CSPH or decompensated cirrhosis
Transplant criteria	UCSF	Milan or 5-5-500 criteria (≤5 cm, ≤5 tumors, AFP ≤500 ng/mL)	Milan however acknowledges variation in practice, particularly in LDLT	Milan	Milan
Role of ablation	Curative therapy for BCLC 0/A disease if resection is not suitable	CP A/B cirrhosis with tumor size 3 cm or less and 3 or less tumors.	Alternative first-line treatment for tumors <3 cm in CPA or B patients	Curative therapy for BCLC 0/A if not for surgery (resection/LT)	Curative therapy for BCLC 0/A if not for surgery (resection/LT)
Role of TACE	First-line therapy in BCLC B disease Bridging therapy	Hypervascular HCC, CP A/B, 2–3 tumors >3 cm OR ≥4 tumors	Unresectable HCC without vascular invasion or extrahepatic spread	First-line therapy in BCLC B disease Bridging therapy	First-line therapy in BCLC-B disease
Role of SIRT	Select patients with intermediate or locally advanced disease	Not mentioned	Alternative to TACE	Bridging therapy BCLC-B and BCLC-C disease	Alternative to TACE for BCLC-B
Role of SBRT	Local tumor control in suitable patients	Not mentioned	Not recommended other than for symptomatic bony metastases	Not recommended pending further evidence	Alternative to ablation in BCLC-A patients who are not candidates for resection
Role of systemic therapy	BCLC-C disease or BCLC-B disease not suitable for locoregional treatment	CPA and good performance status with unresectable HCC not amenable to locoregional treatment	Macrovascular invasion or extrahepatic metastasis. CPA or CPB with caution.	BCLC-C disease or earlier stages progressing/unsuitable for locoregional treatment	BCLC-C disease or BCLC-B disease not suitable for locoregional treatment or adjuvant therapy as above
First-line systemic therapy	Sorafenib or lenvatinib	Bevacizumab + atezolizumab	Sorafenib	Sorafenib or lenvatinib	Bevacizumab + atezolizumab OR Durvalumab + tremelimumab
Second-line systemic therapy	Not specified. Should be offered if preserved liver function and good ECOG PS	Sorafenib or lenvatinib	Regorafenib	Regorafenib	Sorafenib or lenvatinib (if not suitable for clinical trial)
Third-systemic therapy	Not mentioned	Cabozanatinib or regorafenib or ramucirumab	Not mentioned	Not mentioned	Cabozanatinib or Regorafenib or Ramucirumab (if not suitable for clinical trial)
BCLC-D management	Symptomatic care, support care services	Not mentioned	Not mentioned	Symptom management, nutrition, palliative care	Advance care planning

Abbreviations: AASLD, American Association for the Study of Liver Diseases; AFP, alpha-fetoprotein; APASL, Asia Pacific Association for the Study of the Liver; ATSI, Aboriginal and Torres Strait Islander; BCLC, Barcelona Clinic Liver Cancer; CEUS, contrast-enhanced ultrasound; CP, Child-Pugh class; CSPH, clinically significant portal hypertension; EASL, European Association for the Study of the Liver; ECOG, European Cooperative Oncology Group; FLR, functional liver reserve; GESA, Gastroenterological Society of Australia; JSH, Japanese Society of Hepatology; LDLT, living donor liver transplant; LI-RADS, Liver Imaging Reporting and Data System; LT, liver transplant; MDT, multidisciplinary team; PAGE-B, Platelet Age Gender–HBV; SBRT, stereotactic body radiation therapy; SIRT, selective internal radiation therapy; TACE, transarterial chemotherapy embolization; UCSF, University of California, San Francisco.

### Overview of QIs

QIs are useful in clearly defining discrete markers of quality care. QIs can be used to monitor the quality of care delivery both across sites and within a site over time—and thereby aim to identify opportunities for improvement and optimization of the existing systems in place at an institution. Quality improvement in this way should be expected to ultimately improve outcomes for patients in the long term.^19^ In the published academic literature, there are currently only 2 existing statements documenting QIs in HCC,^17^,^18^ both developed using a modified Delphi approach involving large multidisciplinary expert groups from different centers. There is a further statement developed by the National Health Service (NHS) Scotland that has been published online.^20^ NHS Scotland QIs are currently audited, and percentage targets for site-specific performance are specified in the document. Table 2 summarizes the themes across the 3 QI sets.

**TABLE 2:**
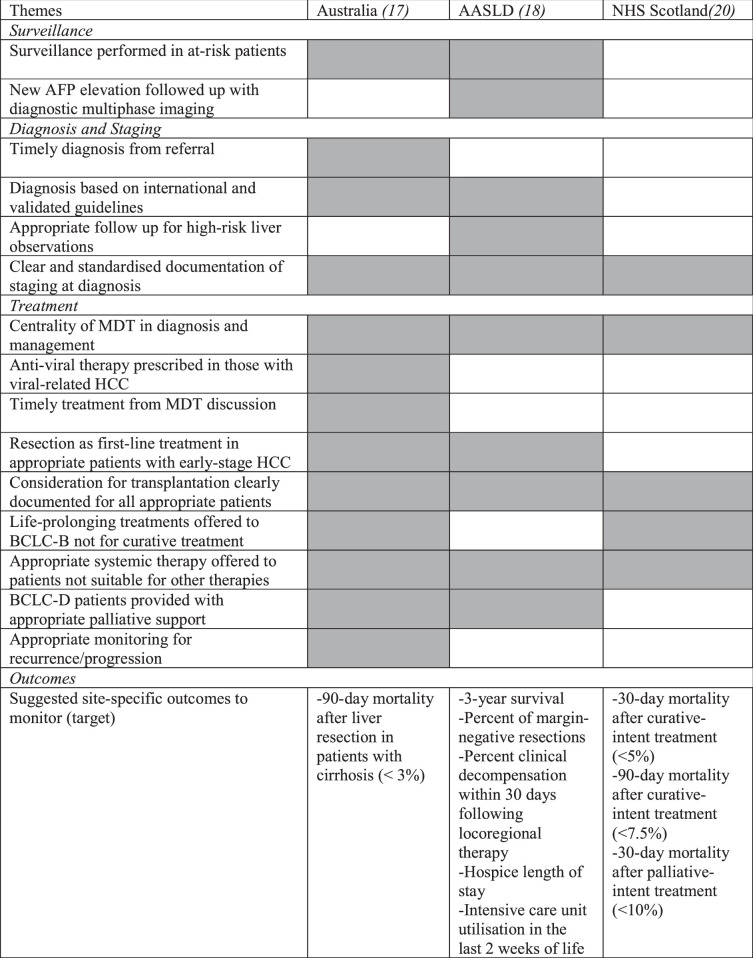
Summary of Quality Indicator Sets

Abbreviations: AASLD, American Association for the Study of Liver Diseases; AFP, alpha-fetoprotein; BCLC, Barcelona Clinic Liver Cancer; MDT, multidisciplinary team; NHS, National Health Service.

### HCC surveillance

HCC in stage Barcelona Clinic Liver Cancer (BCLC) 0/A is amenable to curative therapy but is usually asymptomatic. HCC surveillance is key in identifying HCC in its early stages in order to allow for cure, in contrast to diagnosis in the symptomatic advanced stage where treatment options are limited, and HCC is often fatal. Indeed, HCC surveillance has been shown to be key in reducing HCC-related mortality.^21^,^22^ Despite this, uptake of HCC surveillance programs in at-risk patients has remained poor across multiple different regions of the world,^23^ with common barriers to surveillance, including socioeconomic disadvantage, cultural barriers, lack of centralized surveillance programs, and limited access to subspecialty care.^24^

All 3 western guidelines^8^,^9^,^10^ agree on the need for HCC surveillance in patients with cirrhosis who would be candidates for curative therapy and in select patients with chronic hepatitis B. Unlike the EASL and AASLD guidelines, the GESA guidelines do not include Caucasian patients with Platelet Age Gender–HBV scores of ≥10. A Platelet Age Gender–HBV score of ≥10, which has been validated in Caucasian populations, predicts a 5-year incident HCC risk of >3%,^25^ which translates to greater than the 0.2% annual risk threshold that corresponds to cost-effectiveness.^26^ Western and APASL guidelines agree on the role of 6 monthly ultrasound in surveillance, while Japanese guidelines recommend 3–4 monthly ultrasound in patients with viral cirrhosis and 6 monthly only in those who have nonviral cirrhosis or noncirrhotic chronic viral hepatitis. The addition of tumor marker monitoring to semiannual ultrasound surveillance is recommended by AASLD and JSH guidelines, not recommended by EASL guidelines, and left open to clinician preference in the GESA and APASL guidelines. Adding alpha-fetoprotein (AFP) to ultrasound is a low-cost intervention that has been shown to increase the sensitivity of surveillance for early-stage HCC from 45% to 63%.^27^

The relevant proposed Australian QI is that in patients with known cirrhosis and who are managed through a specialist center, there is documented regular surveillance with ultrasound and/or appropriate alternative liver imaging performed within 6–12 months before the first detection of HCC.^17^ This QI is importantly feasible to audit, as all patients diagnosed with HCC who have a history of management at a specialist center as a patient at-risk for HCC can be retrospectively assessed for prior screening. Significant numbers of patients being diagnosed with HCC without documented recent surveillance would then highlight that patient adherence to recommended surveillance was a potential area of improvement at that center.

In contrast, the AASLD QIs relating to surveillance reiterate surveillance guidelines, specifying populations in which HCC surveillance is recommended, such as those with cirrhosis and noncirrhotic chronic hepatitis B who are at risk based on family history, age, and ethnicity.^18^ Auditing these as QIs would be difficult in the Australian setting, where health care systems are lacking in being able to easily define and capture all patients who are at risk for HCC in order to audit the proportion who have failed to undergo regular surveillance.

### Diagnosis

The Australian QI relevant to diagnosis describes the need for diagnosis confirmation with either established imaging criteria such as Liver Imaging Reporting and Data System (LI-RADS) or with histopathology—with histopathology required to make the diagnosis in all patients where LI-RADS criteria cannot be applied.^17^ Importantly, the LI-RADS algorithm can only be used in adult patients with chronic HBV or cirrhosis, where the cause of cirrhosis is not a vascular disorder or congenital hepatic fibrosis as these conditions can produce atypical imaging findings. It is important that we can be confident that all diagnoses of HCC are accurate, and this QI aims to ensure that adequate documentation is present to establish the diagnosis without any doubt. Additionally, the treatment of LI-RADS 4 subcentimeter lesions with arterial phase hyperenhancement and washout as HCC has been well described in real-world practice despite the recommendation to survey these lesions over the short term, as many are regenerative nodules.^28^ Such deviations from evidence-based care are important to document. Records of patients with HCC can be easily examined for evidence of adequate documentation regarding diagnosis, allowing this QI to be easily audited.

The AASLD QIs similarly affirm that LI-RADS criteria can be used to diagnose HCC in patients with cirrhosis or chronic hepatitis B and specify that LI-RADS-3 lesions should undergo follow-up imaging within 6 months and LI-RADS-4 lesions should have appropriate follow-up determined by a multidisciplinary meeting (MDM).^18^ In an Australian setting, it would be difficult to directly audit all LI-RADS 3 lesions, as many of these are seen in decompensated cirrhosis without HCC and therefore diagnosed outside of a HCC MDM, potentially meaning they would not be readily available to include in an audit focused on HCC care. NHS Scotland QIs include the need to see that a CT or MRI had been performed prior to the first HCC treatment administered—which in practice, should be a certainty.

### Staging

Auditing the appropriateness of treatment allocation in HCC care is entirely based on the accuracy and completeness of relevant staging information in the medical record. As such the quality of documentation regarding HCC staging is a key QI, as it is foundational to assessing all other QIs relating to treatment provision. Clinician’s documentation of cancer staging has historically been described as poor, with >50% of patients with incomplete staging documentation,^29^,^30^ although no such studies have focused on HCC.

Australian QIs regarding HCC staging include documentation of the following: tumor size, number and location of lesions, metastases, vascular invasion, European Cooperative Oncology Group performance status, cirrhosis status, underlying liver disease etiology, assessment of liver function, and overall BCLC stage.^17^ These factors are all feasible to audit via review of documentation at first and subsequent MDT discussions as well as the first clinical review. If deficiencies were noted in the capturing of one or more of these areas, it would be relatively easy to identify and address.

AASLD QI similarly states that tumor burden, liver function, and performance status should be documented at the time of diagnosis of HCC.^18^ NHS Scotland QI states that the following information should be completely documented at diagnosis with a benchmark target set at >90%: BCLC stage, number of liver lesions, size of the largest liver lesion, vascular invasion, cirrhosis status, cause of chronic liver disease, Child-Pugh score, and serum AFP.^20^ The only element included here that is not included in the Australian QI is the serum AFP, which is particularly relevant with respect to transplant eligibility. In the Australian setting, not all patients undergo AFP testing before their initial clinic visit and/or MDTM, and therefore this information may be unavailable at the time of their initial MDT discussion.

### Multidisciplinary care

The use of an MDT as the basis for all major treatment decisions in HCC has been shown to have a marked positive impact on outcomes, including survival.^6^,^7^,^31^,^32^,^33^ Western and APASL guidelines highlight the centrality and importance of MDT in the diagnosis and management of HCC and recommend it as the standard of care.

The Australian QI set clearly cements the need to ensure all patients with HCC are discussed in an MDT meeting—with the expectation that the diagnosis, staging, and treatment planning of a patient with suspected or proven HCC is managed by an MDT.^17^ To capture patients with HCC who are not managed by an MDT, Australian centers may choose to audit patients receiving HCC treatment (resection, ablation, transarterial chemotherapy embolization [TACE], etc.) to ensure that all had been discussed by an MDT prior to receiving their therapy. Cementing this as a standard of care provides additional encouragement to individual providers, including those in more peripheral centers, to refer patients for MDT discussion before commencing treatment locally. Improvement and streamlining of referral pathways are important areas of focus for health policy, in order to ensure timely access to care for all patients regardless of geographic location.

NHS Scotland QI, with a site-specific benchmark target of 95% of patients, also states that all patients with HCC should be discussed in an MDM before commencing treatment.^20^ AASLD QI emphasizes the need not only for patients to be discussed in an MDM but also for the treatment recommendations to be adequately documented in the medical record.^18^

### Surgical resection

Surgical resection, together with percutaneous ablation, are the principal first-line curative therapies for patients with limited disease. Multiple international studies, however, have shown that up to 40% of patients with BCLC 0/A HCC fail to receive upfront curative-intent therapy.^13^,^14^,^15^,^16^

All 3 western guidelines^8^,^9^,^10^ implicitly present resection as the superior treatment modality for achieving durable local tumor cure, in keeping with recent findings of an Australian multicenter cohort study.^34^ In contrast, APASL and JSH guidelines encourage the use of ablation as equivalent to resection in patients with tumors <3 cm,^11^ with the Japanese SURF trial^35^ having shown similar outcomes between the 2 modalities.

EASL guidelines highlight the need for individualized patient assessment based on liver function, portal hypertension, extent of hepatectomy, expected volume of the future liver remnant, performance status, and patient’s comorbidities in deciding on resection versus ablation for an attempted cure. GESA and AASLD guidelines recommend resection in all patients without cirrhosis and in patients with cirrhosis without clinically significant portal hypertension, compensated liver function, and anticipated adequate functional liver reserve post-resection.

While western guidelines emphasize the need for localized disease and compensated liver function to consider resection, APASL and JSH guidelines take a far more individualized approach, considering all patients potentially resectable, even those with more significant liver disease, multinodular tumor and macrovascular invasion.^11^,^12^,^36^ With a focus on maximizing survival even in patients at high risk for further recurrence, and the very limited availability of liver transplant, HCC experts in Asian countries believe that the narrow scope for resection in Western guidelines is not fit for purpose in their clinical practice.^11^,^37^

Australian QI advocates that liver resection should be offered as first-line therapy in patients with preserved liver function, sufficient liver remnant, and absence of significant portal hypertension (or a valid reason for not undergoing treatment).^17^ In auditing this QI, evidence for a documented reason to not proceed to resection should be sought. This may include non-liver comorbidities or the anatomical location of the tumor. AASLD QI very similarly states that patients with BCLC 0/A HCC without portal hypertension should have surgical resection performed when anatomically possible.^18^

### Liver transplantation

Liver transplantation represents the sole therapy that can provide a cure for both HCC and the underlying liver disease and is associated with superior cancer-free and overall survival compared to resection and ablation.^38^ Equitable access to liver transplantation continues to be an ongoing area of investigation, with some evidence to date of disparities based on conventional social determinants of health.^39^

Both EASL and AASLD guidelines consider transplantation as the curative treatment of choice in patients who are not suitable for resection. In contrast, GESA guidelines consider ablation a reasonable first-line alternative curative therapy in place of resection for small HCCs and would not necessarily recommend upfront transplantation for all patients not suitable for resection. The Transplantation Society of Australia and New Zealand guidelines for liver transplantation reserve transplantation as a treatment for patients whose 2-year life expectancy due to liver disease is <50%, and therefore transplantation is generally only recommended for patients with more significant tumor burden rather than upfront treatment in all BCLC A patients not suitable for resection. However, there has been increasing recognition in Australia that even patients with 2 small HCCs, who may be perceived as curable with ablative therapy, should be referred for transplantation, as transplant-free survival is poor in this population irrespective of the treatment received.^40^ Transplant guidelines differ worldwide for many factors but, most importantly, are influenced by the availability of donor organs, the scarcity of which has led to APASL and JSH guidelines reserving transplantation for those with both decompensated cirrhosis and HCC.

JSH, AASLD, and EASL guidelines encourage the downstaging of tumors to within Milan criteria (single tumor <5 cm or <3 nodules with largest <3 cm) prior to transplant listing, whereas GESA guidelines in 2020 outline that HCC within the University of California (UCSF) criteria (single tumor <6.5 cm or <3 nodules with largest <4.5 cm) is the standard in Australia. Subsequent to GESA guidelines, the most up-to-date Transplantation Society of Australia and New Zealand guidelines have allowed transplant centers to use either the METROTICKET 2.0 “up-to-7” rule, with calculated 5-year post-transplant survival of >70%, or UCSF criteria to determine eligibility for transplant listing. APASL guidelines allow for center flexibility in determining transplant criteria, particularly in those receiving living donor liver transplants.

Access to liver transplantation for HCC remains challenging in Australia, given the relative paucity of donor organs and the ever-present challenge of correctly prioritizing patients with HCC on the transplant waitlist alongside those with end-stage liver disease who have higher short-term mortality while also minimizing the known risk of waitlist dropout and post-transplant HCC recurrence.^41^ Inequalities in access to liver transplantation, driven by variations in referral patterns as well as disparate geographic organ availability, have been well described in the literature,^42^,^43^,^44^,^45^ with factors, such as distance from the transplant center, socioeconomic status, liver disease etiology, age, gender, ethnicity, and cultural beliefs all playing an unconscious role in biasing medical decision-making and subsequent access to liver transplantation. There is therefore a clear need to audit the adherence to treatment guidelines regarding transplant referral.

Australian QI reflects that in all patients with HCC within transplant criteria who are not suitable for curative hepatic resection or ablative therapy, there should be a documented discussion of liver transplantation.^17^ This is relatively simple to audit, with a review of clinical notes to ensure that there is a valid, consciously considered reason for not referring the patient if they otherwise appear suitable based on tumor burden and performance status. Importantly, however, this QI would fail to capture under-referral for other patients who would clearly benefit, such as those with 2 small HCCs or those who developed a second distinct tumor during follow-up.

AASLD QIs take a more absolute approach, with all patients with HCC without extrahepatic disease who are not resection candidates and without absolute contraindications for liver transplant, expected to be referred for transplantation.^18^ This QI includes significantly more patients than the Australian QI. Similarly, the NHS Scotland QI suggests that all patients who meet UK transplant listing criteria (within Milan criteria or single tumor 5–7 cm with no significant progression over 6 mo) are referred to the Scottish Liver Transplant Unit for opinion and management.^20^ This reflects differences in transplant practices, with living donor liver transplantation offered overseas for such patients but not in Australia. Such an approach is unlikely to be feasible in the Australian setting, where transplant centers would not be equipped to deal with such a large volume of referrals. Furthermore, there is a clear role for hepatologists at nontransplant centers to differentiate suitably between patients qualifying for transplant referral as per Australian guidelines and those who do not.

### BCLC-B disease

TACE is known to improve survival in patients with BCLC-B HCC, with relatively low complication rates in appropriately selected patients.^46^ Despite this, it is a therapy that is potentially underutilized, with international studies demonstrating up to 36% of patients with BCLC B fail to receive TACE.^13^,^14^,^15^,^16^ The role of TACE in downstaging or bridging patients with BCLC A/B disease to curative liver transplantation has been well established.^47^,^48^ However, for all patients, whether transplant-eligible or not, all guidelines recommend TACE as the treatment of choice in BCLC-B patients not suitable for upfront curative therapy due to the clear survival benefit.^8^,^9^,^10^ Australian QI therefore suggests that TACE (or alternative therapies such as selective internal radioembolization therapy) is offered to patients with BCLC-B HCC not suitable for curative treatment with the intent to delay progression and prolong survival, and in a select subset of patients, down-stage tumor burden to facilitate future curative therapy.^17^ An audit of all patients with BCLC-B would hope to identify the overall proportion of patients who fail to receive appropriate treatment and potentially the underlying reasons for this. NHS Scotland QI aims to broadly assess the proportion of patients not receiving curative therapy who receive some form of life-prolonging treatment such as TACE, radiotherapy, or systemic therapy.^20^ They set a relatively low target of 40%, as their QI effectively includes those with BCLC-B, C, and D disease, many of whom would not be suitable for treatment.

It should be noted that stereotactic body radiation therapy and selective internal radioembolization therapy have both been shown to result in excellent outcomes in BCLC-B disease, with a subset of patients with long-term responses seen.^49^,^50^ With further randomized controlled trial evidence, stereotactic body radiation therapy, and selective internal radioembolization therapy may find a more prominent role in clinical practice, with guidelines and QIs to reflect this. Additionally, combination locoregional and systemic therapy has been shown to have synergistic clinical benefits^51^ and is the subject of ongoing research.

### BCLC-C disease

While all guidelines recommend systemic therapy as the treatment of choice in BCLC-C disease,^8^,^9^,^10^ only 20.1% of patients managed in the Asia Pacific region actually receive systemic therapy, with others receiving no treatment (23.9%) and others receiving alternative treatment modalities inconsistent with accepted international guidelines (61.8%).^52^ Proposed Australian QIs include that in all patients with BCLC-C disease, systemic therapy is offered with the intent to prolong survival and in particular, that currently approved first-systemic therapy is offered to eligible patients.^17^ Because bevacizumab/atezolizumab combination immunotherapy has been shown to offer superior overall and progression-free survival compared to the oral multikinase inhibitors, it is important that auditing of Australian patients with BCLC-C HCC examines the relative proportions of those receiving first-line systemic therapy, alternative systemic therapy, nonevidence-based treatment and best supportive care. AASLD QIs similarly specify that patients with BCLC-C HCC should be offered systemic therapy, and more broadly, that all patients who are not candidates for surgery or locoregional therapy should be considered for systemic therapy.^18^

### BCLC-D disease

Palliative care involvement has been shown to improve patient quality of life and reduce health care utilization in patients with end-stage liver disease^53^,^54^; however, there is a paucity of HCC-specific literature. Nonetheless, Australian guidelines emphasize the importance of early palliative care involvement, with the understanding that palliative care medicine provides a valuable role in holistically managing physical, psychological, and spiritual needs and addressing the concerns of patients and their families.^55^,^56^ Australian QIs reflect this with a target of all patients with BCLC-C or D disease referred to palliative care and the need for all patients with BCLC-D disease to be offered symptom management in conjunction with supportive care services.^17^ This is an important area to focus on, as historically palliative care has been underutilized in this patient cohort.^57^ AASLD QIs similarly involve auditing those with BCLC-D disease receiving palliative support and highlight the need for advanced care planning in BCLC-C/D HCC.^18^ Advanced care planning, or advanced care directive, has historically been poorly implemented in patients with liver disease but is known to improve the quality of end-of-life care and reduce inappropriate or futile medical interventions.^58^ This is a potential additional area that could be useful to audit in the future in the Australian context.

### Site-specific outcomes

Site-specific outcomes can be monitored prospectively to assess the quality of care over time and allow for early identification of underperforming centers so that any issues can be addressed. The only proposed site-specific outcome in the Australian QI set is the perioperative 90-day mortality after liver resection in patients with cirrhosis, with an expected standard of less than 3%.^17^ Higher 90-day mortality rates than this would raise concern primarily for inappropriate patient selection but also local surgical performance below the modern standard of a tertiary referral center. The figure of 3% for perioperative mortality was first proposed in 2012 by a panel of experts from EASL and the European Organisation for Research and Treatment of Cancer based on low perioperative mortality figures reported by multiple centers^59^,^60^,^61^ corresponding with advances in surgical techniques. In the Australian setting, perioperative mortality is routinely audited by surgical units, and this QI can be very feasibly implemented in routine clinical practice.

NHS Scotland QIs similarly aim to audit short-term mortality after resection, transplantation, ablation, and TACE.^20^ Thirty- and 90-day mortality are reported according to specific curative-intent treatment modality (liver transplant, resection, and ablation) with an expectation that 30-day mortality is <5% and 90-day mortality <7.5%.^20^ Thirty-day mortality is reported alone for patients receiving TACE with an expected benchmark <10%.^20^

AASLD QIs include a large number of outcome data, including 3-year survival, percent of margin-negative resections, percent clinical decompensation within 30 days following locoregional therapy, length of stay in patients admitted to hospice, and intensive care unit utilization in the last 2 weeks of life.^18^ Three-year survival is useful to monitor but is unlikely to perform well as a QI in the Australian context. Patients with HCC are an extremely heterogenous group across BCLC stages, and multiple factors play into survival at 3 years beyond quality of care. Additionally, patient loss to follow-up would likely significantly limit the practical utility of this QI. Clinical decompensation at 30 days following locoregional therapy, like 90-day mortality postsurgical resection, likely serves as a surrogate for the quality of patient selection and technical expertise in delivering TACE to borderline candidates. Similar outcomes could be audited in the Australian context, perhaps with a focus on the need for readmission within 30 days. Length of hospice stay is of limited benefit as a QI in the Australian context, where very few patients are referred for inpatient palliative care in general, let alone prematurely.^56^ Intensive care unit utilization within the last 2 weeks of life, in principle, provides a surrogate assessment of the appropriateness of goals of care and implementation of advance care planning but in reality, would be nuanced and difficult to interpret. Patients with BCLC 0/A/B HCC may present with acute-on-chronic liver failure due to their underlying cirrhosis, and if they are candidates for curative therapy and have evidence of a reversible precipitant, intensive care unit admission for supportive care is warranted as many patients will recover with appropriate care.^62^ Such patients may still be at risk of significant short-term mortality, and this would confound this proposed QI, particularly in transplant centers where high numbers of waitlisted patients with both decompensated cirrhosis and HCC within transplant criteria may frequently require intensive care unit admission.

### Future directions

CQRs are organizations that monitor the appropriateness and effectiveness of health care by routinely collecting, analyzing, and reporting information about treatment and outcomes in order to continually identify areas for improvement.^63^ With the variation in care that has been well described in HCC,^52^,^64^,^65^ we believe that the implementation of a national HCC CQR is of crucial importance to standardize and maximize the quality of care. Such a CQR would ideally be working in direct partnership with the Commonwealth as well as all Australian states and territories and would be available to all Australian HCC centers for participation on an opt-in basis.

We propose that this CQR would function to monitor rates of achievement of the 23 QIs proposed in the recent Australian QI set,^17^ which we believe to be both valid indicators of quality of care and feasible to monitor. Other groups have described the development of metrics that are able to self-calculate through a direct interface with the existing electronic medical record.^66^ We propose that we build such systems directly into the CQR, and allow appropriate software packages to automatically calculate rates of attainment of QIs directly out of the data registry. Such metrics can be monitored directly by the individual sites and centrally. Suboptimal site performance on any one particular QI can therefore be recognized early and appropriately addressed. For many of the QIs relating directly to guideline adherence or adequacy of documentation, we propose the use of care bundles or pro-forma as day-to-day standardized reminders of appropriate evidence-based care and documentation. Such measures have been shown to reduce variation in care and improve rates of guideline adherence in multiple different areas of clinical practice,^67^ although no such evidence exists specifically for HCC care. For other QIs, a targeted site-specific audit is needed to understand the reasons for suboptimal performance as the reasons for deficiencies may be nuanced, multifaceted, and unique to the managing center. The utility of the CQR, in this case, is to facilitate earlier identification than would occur otherwise. Identification of tailored, appropriate, evidence-based quality improvement initiatives, such as those listed in Table 3, may then be applied.

**TABLE 3:** Summary of evidence-based quality improvement initiatives in HCC care

Quality metrics	Evidence-based interventions
Surveillance	-Centralization of surveillance^68^ -Mailed outreach invitations^69^ -Primary care physician training or reminders^70^,^71^ -Nurse-led surveillance programs^72^
Diagnosis and staging	-Standardized note templates^a^ ^73^
Treatment	-Decision-making through multidisciplinary team meeting^6^,^7^ -Multispecialist care^6^,^74^ -Consolidation of surgery with known volume-outcome relationship at higher-volume centers^a^ ^75^ -Use of expanded liver transplant criteria rather than Milan^76^ -Automated electronic medical record prompts for palliative care referral^a^ ^77^

^a^ Evidence in general oncology, no direct evidence in HCC.

In addition to the 23 QIs produced by the Australian group of experts,^17^ we believe it would be useful to monitor a broader range of site-specific and treatment-specific outcomes as described in the American^18^ and Scottish QIs,^23^ including 30 and 90-day complication, readmission, and mortality rates following resection, ablation, TACE, and other specific locoregional treatments. Outcomes would be made available directly to sites via a benchmarking process involving the calculation of the national average with 95% CI,^78^,^79^ to identify significant unwanted variation from the expected standard for select clinical indicators. Underperforming centers would then have the opportunity to undertake site-specific audits to identify areas for improvement, such as patient selection, technical factors, and operator performance. In the event of persistent underperformance at a particular center, consideration could be made for consolidation of treatments at low-volume centers to be redirected to higher-volume centers. Evidence out of the United States, for example, has shown that survival outcomes after hepatic resection are superior at centers performing more than 10 resections a year compared to lower volume centers^80^; however, these findings have not been reproduced in other populations,^81^ and no such studies have been done in Australia.

With the expected advancements in HCC management, corresponding updates in treatment guidelines, and improving expected outcomes, QIs can be revisited and revised over time according to new evidence, allowing the utility of the CQR to remain up-to-date in the long term. With continual reassessment of QIs and appropriate early recognition of outlier rates of QI achievement and/or site-specific outcomes, the proposed national CQR can be expected to be of benefit even in the long term.

## CONCLUSIONS

With the increasing burden of HCC, the complexities around management, and the high lethality of the disease, there is a clear and urgent need to maximize the quality of care to optimize patient outcomes. The implementation of a national CQR to prospectively monitor guideline adherence, rates of achievement of validated QIs, and site-specific outcomes is likely to translate to clinical benefits for all Australian patients by identifying and addressing adverse variations of care. Such a program would serve as a model for other countries looking to improve their HCC outcomes at a national scale.
